# Can a Biodegradable Implanted Bilayered Drug Delivery System Loaded with BMP-2/BMP-12 Take an Effective Role in the Biological Repair Process of Bone–Tendon Injuries? A Preliminary Report

**DOI:** 10.1155/2017/7457865

**Published:** 2017-06-04

**Authors:** Baran Komur, Yener Akyuva, Numan Karaslan, Mehmet Isyar, Seyit Ali Gumustas, Ibrahim Yilmaz, Semih Akkaya, Duygu Yasar Sirin, Cagri Ata Mutlu, Ahmet Guray Batmaz, Olcay Guler, Mahir Mahirogullari

**Affiliations:** ^1^Kanuni Sultan Süleyman Training and Research Hospital, Department of Orthopaedics and Traumatology, 34303 Istanbul, Turkey; ^2^Ministry of Health, Department of Neurosurgery, 59100 Tekirdag, Turkey; ^3^Namik Kemal University School of Medicine, Department of Neurosurgery, 59030 Tekirdag, Turkey; ^4^Acibadem Hospitals Group, Department of Orthopaedics and Traumatology, 34752 Istanbul, Turkey; ^5^Ministry of Health, Dr. Lutfi Kirdar Training and Research Hospital, Department of Orthopaedic Surgery, 34865 Istanbul, Turkey; ^6^Department of Medical Pharmacology, Istanbul Medipol University School of Medicine, 34810 Istanbul, Turkey; ^7^Private Denizli Surgery Hospital, Department of Orthopaedics and Traumatology, 20070 Denizli, Turkey; ^8^Faculty of Arts and Sciences, Department of Molecular Biology and Genetics, Namik Kemal University, 59030 Tekirdag, Turkey; ^9^Department of Medical Sciences, Acıbadem University School of Medicine, 34752 Istanbul, Turkey; ^10^Memorial Health Group, Department of Orthopaedics and Traumatology, 34758 Istanbul, Turkey; ^11^Medical Park Bahcelievler Hospital, Department of Orthopaedics and Traumatology, 34180 Istanbul, Turkey

## Abstract

**Background:**

Use of biodegradable and biocompatible materials in the orthopedic surgery is gaining popularity. In this research, the rate of controlled release of a bilayered prototype biomaterial designed to promote osteoblastic and tenoblastic activity was calculated using pharmacochemical methods.

**Methods:**

The first part of the design, composed of a sodium tetraborate, polyvinyl alcohol, and starch based hydrogel, was loaded with bone morphogenic protein-2. The second part which was composed of a sodium tetraborate, polyvinyl alcohol, and chitosan based hydrogel was loaded with bone morphogenic protein-12. Osteochondral and tendon tissue specimens were obtained from patients with a diagnosis of gonarthrosis and primary bone cells and tendon cells cultures were prepared following treatment with collagenase enzyme. Cell samples were collected from the groups by means of an invert light microscope and environmental scanning electron microscope underwent at the 1st and 21st days. The level of osteogenic differentiation was measured by the activity of alkaline phosphatase. For the statistical evaluation of the obtained data, groups were compared with post hoc Tukey test following analysis of variance. Level of significance was accepted to be <0,01.

**Results:**

Both osteogenic and tenogenic stimulation were observed in the cultured specimens. In comparison to the control groups, the rate of proliferation of healthy cells was found to be higher in the groups to which the design was added (*p* < 0.01).

**Conclusions:**

Our research is a preliminary report that describes a study conducted in an in vitro experimental setting. We believe that such prototype systems may be pioneers in targeted drug therapies after reconstructional surgeries.

## 1. Introduction

A bone fracture avulsion may occur in some injuries to the area where tendon cleaves into bone. Recovery occurs after bone union with the tendon attachment. Nonetheless, a full recovery may not occur even after appropriate rehabilitation in such cases. As a result, movement restrictions may arise that depend on power loss and immobilization duration [1]. It is known that bleeding and hematomas occur in fracture areas as a result of periost and soft tissue damage in blood vessels and bone marrow during trauma. There are some studies that focus on how a fracture hematoma affects fracture healing. It is thought that growth factors, thrombocytes, and other proteins released from other cells in fracture hematomas lead to cell migration, proliferation, and matrix synthesis in fracture healing [2–4].

On the other hand, pluripotent mesenchymal cells provide formation of fibrous tissue, cartilage, and bone probably by common origin in fracture areas [5]. Whereas some of these cells originated from damaged tissues, others reach the area through blood vessels. Osteoblast cells originate from the periost cambium layer. Therefore, periosteal cells play an important role in cartilage, especially in childhood, because of its structure. Periost is thicker and rich in cells. With increasing age, periost becomes thinner, and its function in the healing of osseous tissues decreases. Osteocytes originated from the endosteal surface do not participate in forming repair tissues. During bone healing, granulation tissue substitutes with hematomas and most of the cells responsible for osteogenesis can be detected in this granulation tissue [3, 4, 6].

The rate of biomediators and oxygen in the area affects cell function in the repair process. Biomediators play a part in cell interaction, cell division, and matrix synthesis and tissue differentiation. They bind to specific receptors in target cells and trigger the signal transmission system. This signal forms a biological response after reaching the cell nucleus. Afterwards, a range of protein syntheses start. One of these proteins is bone morphogenic protein- (BMP-) 2, a protein growth factor that triggers signal transmission and induces osteoblast formation. Another protein growth factor, BMP-12, activates tenoblasts [7, 8].

Current treatment modalities to improve osteogenesis, chondrogenesis, and tenogenesis include conventional injections of many growth factors in a protein/peptide structure, such as BMP-2, BMP-12, and/or platelet-rich plasma [9–11]. However, in these treatment modalities, intra-articular injections of growth factors were not superior to viscosupplementation in terms of tissue repair [12, 13]. Protein and/or peptide growth factors have a very short half-life and lose their bioactivity in seconds when applied without a drug delivery system [14–17].

In this study, in order to increase proliferation of bone and tendon cells, an injectable hydrogel was designed as a drug delivery system for BMP-2/BMP-12. We aimed to prolong the half-life of BMP-2/BMP-12 and maintain their biofunctional features.

Upon reviewing the literature, we found no studies focusing on the treatment of both tendon and bone tissues using hydrogel containing BMP-2 and BMP-12, which can be applied directly to bone and damaged tendon areas. Our results showed that, with our novel design, hydrogel proliferation of osteoblasts and tenoblasts was induced. For this reason, we believe that our study will contribute to the literature.

## 2. Materials and Methods

Media used for primary bone cell cultures were prepared freshly and used for cell viability, toxicity, and proliferation analyses and changed every other day. The researchers who carried out molecular analyses were not informed about which hydrogel growth factors were embedded. They were blind to the contents of the control and experimental groups. The analyses were repeated at least three times.

### 2.1. Exclusion Criteria for Tissues Used for Cases

Tissues obtained from the cases with Paget, primary/secondary hypo/hyperparathyroidism, micro osteoporosis, acromegaly, primary osteogenic sarcoma, and bone metastasis were not included in the study since they might affect the alkaline phosphatase (ALP) level. Besides, the cases taking drugs that interact with the CYP2A6 enzyme, the p450 system, were excluded from the study. In addition, the cases receiving traditional antirheumatic drugs and biological therapy agents, such as rituximab, etanercept, adalimumab, and abatacept, were excluded [18–20].

### 2.2. Preparation of Hydrogels and Primary Cell Cultures

**Figure 1:**
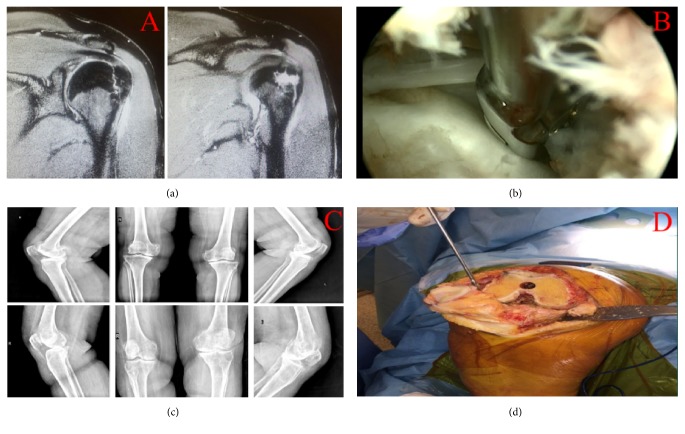
(a) Coronal MRI view of shoulder. (b) Arthroscopic view of shoulder. (c) X-rays of patients that have grade-IV gonarthrosis. (d) A view of knee arthroplasty surgery.

Tissues were obtained from resected bone tissues of the cases who did not respond to medical and conservative treatments, who had advanced osteoarthritis according to Kellgren-Lawrence scoring [21] (average age 67 years; *n* = 6), and who applied total knee arthroplasty used for the purpose of preparing primary osteocyte cultures. Furthermore, because of rotator cuff tear, the cases applied arthroscopic rotator repair. Rotator cuff ends were cut by a punch. These tissues, which are 3-4 mm long, were disintegrated into 1 mm^3^ pieces (Figures 1(a)–1(d)).

Both bone and tendon tissue pieces were transferred into different dishes containing 5% penicillin-streptomycin and Dulbecco's modified eagle's medium (DMEM). Afterwards, they were transferred into the laboratory at 4°C. Tissues were washed by phosphate buffer saline (PBS) solution, and red blood cells were removed in the flow cabinet.

At first, samples were minced mechanically. These tissue samples were shivered enzymatically by adding* Clostridium histolyticum* based collagenase type I (475 *μ*g/mL) and type II (125 5 *μ*g/mL) enzymes, which were solubilized in Hank's balanced salt solution (HBSS). Samples that were taken to 5% CO_2_ at 37.4°C incubation were consecutively centrifuged twice at 1200 rpm for 10 min. on the following day. Cell pellets remaining at the bottom were resuspended by culture media that had been prepared by DMEM. The samples were left for incubation for 72 h.

Freshly prepared media were added to wells and incubated for 21 d by changing their media every other day. Afterwards, osteogenic stimulator media were added every other day for 16 d in order to induce osteoblastic activity [22]. Alpha-modified minimum essential medium was added and used to feed the samples in tendon cell cultures. Human primary bone cell cultures were taken into the experiment, which reached minimum 90% confluency.

The cells were counted using Thoma Lam and Trypan blue. Some 3.7 × 10^4^ osteocytes and 4.6 × 10^6^ tenocytes were placed in 24-well plates for further experiments.

Two-layer hydrogel was prepared to induce mineralized matrix formation, in order to be used against bone–tendon injuries to increase healthy proliferation and osteogenic/tenogenic effects. BMP-2 was added to the first layer, and BMP-12 was added to the second layer of hydrogel. In this manner, following biodegradation, BMP-2 and BMP-12 were released consecutively.

**Figure 2:**
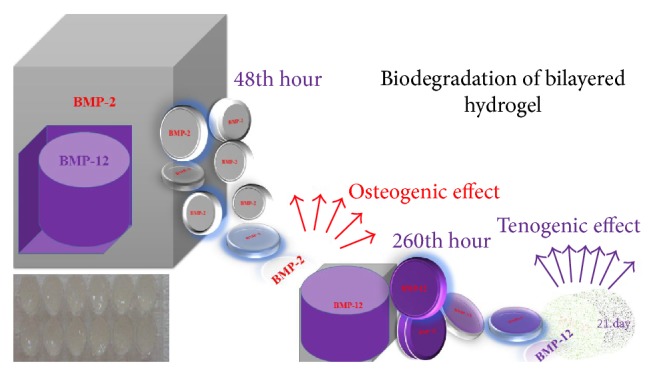
Illustration of biodegradation of bilayered drug delivery system.

BMP-2 (10 ng/mL) was added to the first layer at the rate of 1 : 3, which was prepared by the mixture of sodium tetraborate and PVA starch for the purpose of adjusting biodegradation. On the second layer, sodium tetraborate and PVA-chitosan were prepared at the rate of 2 : 3, and BMP-12 (10 ng/mL) was added. The mixtures were prepared separately and homogenized. Weak crosslinks created with PVA and starch, with the addition of sodium tetraborate and an amidon reagent establishing the high-density crosslinked network, were provided to release drugs in a controlled way up to 21 d. The hydrogel composing the second layer was embedded into the first layer of hydrogel. A schematic representation of the drug delivery system design is given in Figure 2.

**Figure 3:**
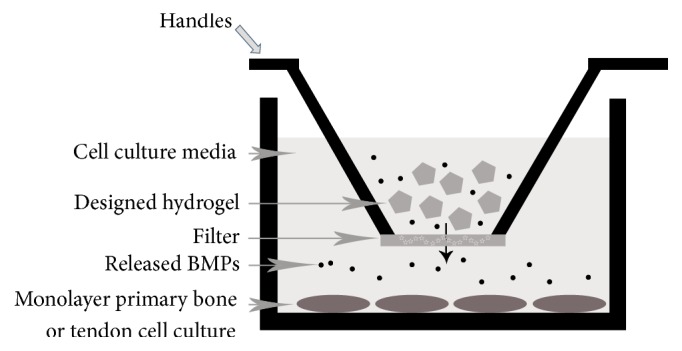
Illustration of representation BMPs application with hydrogel delivery system showing controlled released BMPs migrating from upper chamber to lower chamber through porous filter.

Group I was composed of primary human bone tissues without hydrogel, whereas Group II was composed of primary human bone tissues cocultured with hydrogel. Cell cultures obtained from primary tendon tissue without hydrogel were in Group III. Group IV was composed of primary tendon cell cultures cocultured with hydrogel. Hydrogels prepared in equal volumes were placed on the upper part of the transwell chambers. A schematic representation of the experimental design is given in Figure 3.

The chamber was placed on the monolayer osteocyte/tenocyte culture, and the culture was monitored regularly by changing the nutrient medium every 2 d.

### 2.3. Calculation of Physicochemical Analyses and Controlled Release Amount of the Design

In the present study, a swelling test was performed to calculate molecular weight among crosslinks of swollen hydrogel, based upon Flory Rehenner's theories [16, 23]. In order to calculate the swelling balance in ultrapure water, hydrogel samples were cut into pieces. Their inner diameter was 4.5 mm, and they were 1.5 mm thick and 10 mm long. These pieces waited for 6 h at 37 ± 0.5°C after they had been frozen for 6 h at −20°C.

The dry weights of the samples were recorded. These hydrogel samples waited for 240 min at regular intervals in 25 mL ultrapure water. Samples were taken every 20 min and dried gently using blotting paper, and the wet weights of the samples were recorded. Diameters of the hydrogel samples were calculated using digital calipers after the swelling balance, which made it possible to calculate the time dynamic weight swelling index (hydration percentage) of the drug delivery system in which BMP-2 and BMP-12 were not embedded. The formula was as follows: dynamic weight change (hydration percentage) % = (*W*_2_−*W*_1_/*W*_1_) × 100 [15–17]. *W*_2_ indicates the weight of swollen hydrogen piece in the water, and *W*_1_ indicates the initial dry weight. Release experiments were done in PBS (pH = 7.4). Absorbance changes were calculated at 260–280 nm absorbance and 37.4°C during the release of growth factors, using a UV spectrophotometer.

A PBS solution containing a 0.1 N 0.9% isotonic sodium chloride solution was prepared. The hydrogels containing BMP-2 and BMP-12 waited in the beaker for 8 d, with the PBS solution being changed every 12 h. While changing PBS, one milliliter of PBS sample containing materials released from the hydrogel was analyzed spectrophotometrically. PBS and each of the hydrogel contents were used as a blank for spectrophotometric analysis.

Time graphs were drawn against the absorbance by means of acquired data. From the amounts of BMP-2 and BMP-12, which were initially loaded in the hydrogel, BMP-2 and BMP-12 amounts released into the environment were removed. Thus, growth factors that were absorbed in the hydrogel system and not controllably released into the environment were calculated.

### 2.4. Inverted Light and Environmental Scanning Electron Microscopy

Cell organizations and morphology were evaluated through inverted light microscopy (4x, 10x, 20x, and 40x). For ESEM analysis, the cell culture media were removed, cells were fixed with a 97.5 mL cacodylate buffer, and a 2.5 mL solution containing glutaraldehyde was added to wells covering the samples. After incubation for 2 h at room temperature, samples were washed three times with buffers. Samples were evaluated by ESEM on the 7th and 21st days without being covered with golden or silver stains. FEG ion pumps were used to achieve a high vacuum [15–20, 24].

### 2.5. Measurement of ALP Activities

ALP activity is one of the most frequently used indicators of osteogenesis, as it reflects the ratio of differentiated cells as osteogenic. ALP activity was assigned by p-nitrophenyl phosphate as the substrate standard curve. After phosphatase reaction of p-nitrophenyl phosphate, the osteogenic effect indicators of the samples in the cultures were calculated by ALP activity, which is based on the formation of p-nitrophenolate from p-nitrophenyl.

Samples from the well plates were taken into falcon tubes on the 1st, 7th, 14th, and 21st days in order to examine osteoblastic differentiation, and they were stored at −20°C. At the end of this duration, 1 mol/diethanolamine buffer was added to the falcon tubes in a ratio of 1 : 4 (0.5 mmol/L MCL2), and to the substrate solution (10 mmol/L p-nitrophenyl phosphate) [25]. Analyses were carried out at 415 nm (OD) by a UV spectrophotometer in terms of absorbance.

### 2.6. MTT Cell Vitality, Toxicity, and Proliferation Analyses

Cell viability tests were evaluated after enzymatic disintegration of the tetrazolium ring by dehydrogenase enzymes was completed and blue formazan crystals were formed. MTT analyses were evaluated in accordance with commercial kit bulletins on the 1st, 7th, 14th, and 21st days [15–20, 24, 26].

A culture medium solution containing MTT and DMEM (1 : 6) was added to each well in the amount of 500 *μ*L. Some 25 *μ*L 10% sodium dodecyl sulfate was added to each well after 3-h incubation in order to avoid color reaction. Some 200 *μ*L of these solutions was transferred into 96-well plates. Afterwards, the samples were evaluated in enzyme-linked immunosorbent assay (ELISA) at 540 nm (OD).

Cell viability was calculated in terms of %. The formula was as follows: proliferation = test (OD/control OD) × 100. The number of living cells was considered as 100% on the first day [24]. The number of living cells was reported in % on the 1st, 7th, 14th, and 21st days, and we examined whether there were any differences in proliferation across groups.

### 2.7. Statistical Analysis

Descriptive statistics were shown as mean ± standard deviation. In the analyses of the acquired data, results were evaluated by cell number, MTT cell viability, toxicity, and proliferation. The Minitab R16 program was used in the statistical evaluation. The evaluations were made at a 95% confidence interval.

The results were evaluated using variation analysis (ANOVA) to find whether there were significant differences across the groups. When there were differences across the groups, Tukey's honestly significantly different (HSD), one of the multiple post hoc pairwise comparisons tests, was used to determine the difference and investigate the false positives, so that the different averages across the experiment groups were evaluated. *p* < 0.01 was considered to be highly significant.

## 3. Results

### 3.1. Physicochemical and Pharmacokinetic Evidence of the Design

**Figure 4:**
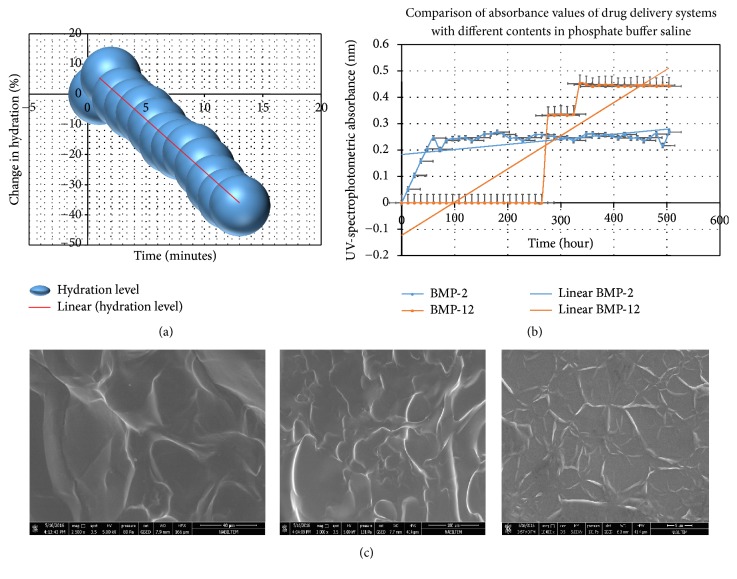
(a) Swelling balance of hydrogel. (b) Expression of absorbance type into amount of released growth factors to loaded design against time. (c) Evaluation of the surface morphology of the hydrogel by ESEM. The images were recorded at a pressure of 219–231 Pa in ESEM vacuum mode, at 1000x–10.000x magnification and 82,9 *μ*m resolution depths (HFW), at an operating voltage of 5.00 kV, and at a working distance of 9,4–10,7 mm.

The hydration change percentage in the drug delivery system in which growth factors were not loaded and release kinetics of the system in which BMP-2 and BMP-12 were embedded was determined (Figure 4(a)). The change of hydration ratio indicates the release from pores of the hydrogel. Statistical evaluation of controlled release of BMP-2 and BMP-12 from the hydrogel reported that the rate of release was maintained at the same level. While the first release of BMP-2 commenced at the 12th hour and the maximum release was recorded at the 48th hour and continued until the 21st day, BMP-12 commenced at the 260th hour and continued until the 21st day (Figure 4(b)).

Spectrophotometric analysis showed that, at the end of the 21st day, the release rates were 89.02%  ±  5.01 (BMP-2) and 83.78 ± 4.32 (BMP-12). The initial values of BMPs loaded into the hydrogel could not be detected. The unreleased amount was presumed to be absorbed within the structure of the hydrogel matrix. Results of a paired-samples test showed that the amount of BMP-2/BMP-12 released into the medium was statistically significant (BMP-2; *p* = 0.008, *t* = −2.18, BMP-12; *p* = 0.003, *t* = −2.11; 95% confidential interval).

SEM analysis of the hydrogel revealed a smooth surface topography due to its high polymeric content. Numerous porous structures, a reflection of the high density of the monomeric content, were also observed (Figure 4(c)).

The interstices within the structure of the hydrogel that could enable controlled release of BMP-2 and BMP-12, a drug with a protein structure, were remarkable. Crosslinking was also found to be very compact.

### 3.2. Evaluation of Culturized Samples by Inverted Light and Environmental Scanning Electron Microscopy

**Figure 5:**
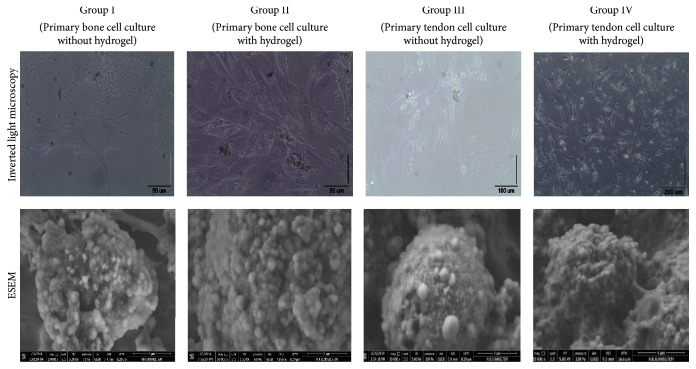
Inverted light microscope and microvisualization of ESEM with cell culture samples and pericellular structures. The images were recorded at a pressure of 219–231 Pa in ESEM vacuum mode, at 1000x–10.000x magnification and 82,9 *μ*m resolution depths (HFW), at an operating voltage of 5.00 kV, and at a working distance of 9,4–10,7 mm.

Normal cell morphology and proliferation were reported before the hydrogel applications (Figure 5).

It was found that cell proliferation was higher in Groups II and IV than in the control groups (Groups I and III). In addition, the extracellular matrix formation was significantly increased on the 21st day in Groups II and IV. In Groups II and IV, it was remarkable that the maturation of cells and mitotic and adhesion activity were increased.

### 3.3. Evaluation of ALP Activity Analyses

**Figure 6:**
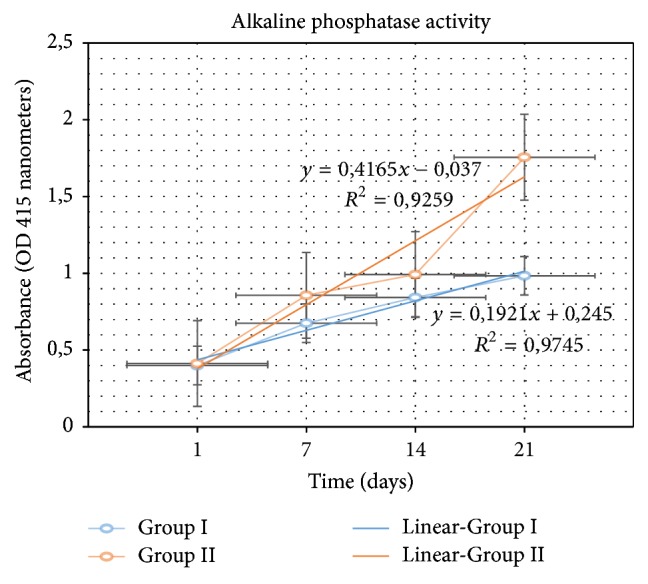
Level of ALP expressed of absorbance type against time.

Increased ALP expressions were detected in Groups II and IV when compared with the control groups. More ALP activity was observed in Group II (Figure 6).

### 3.4. Statistical Evaluation of ELISA Data Acquired from Biochemical Analyses

According to the results of the experiments that had been done on the 1st, 7th, 14th, and 21st days, it was found that cell viability and proliferation, along with osteoblastic activity, were observed the most in Groups II and IV (Table 1).

## 4. Discussion

Interest in both amateur and professional sportive activities has gained momentum in recent years. The fact remains that permanent or temporary bone–tendon injuries in bone tendons occur frequently, resulting from direct lateral strokes to the elbow and falling over the hand(s) [27]. Apart from sportive activities, bone–tendon damage may be the case in geriatric patient populations with low energetic trauma where bone quality is low, or in young patients with high energetic trauma, such as motorcycle crashes [28–32].

Moreover, plate and flexor tendons applied in surgery may both irritate extensor tendons and form extensor pollicis longus rupture. Following nondisplaced fractures, compression in extensor mechanisms may occur, which may lead to mechanical compression in tendons and rupture after the formation of local ischemic areas [33, 34]. Apart from these, damage may occur in both the upper and lower extremity Achilles bone–tendon junction [35].

Following these types of damage, it was found that healthy bone, tendon, and cartilage tissues disappeared in time. In order to repair damaged tissues and sustain natural tissues, using drug delivery systems that activate biological repair mechanisms or repair the defective area by biological tissues has been one of the most researched areas recently [15–17, 24, 26, 36–38].

In the present study, we devised a practical design that can be used in surgical operations by double lumen injectors in cases with bone–tendon damage. The design has a mechanism that allows for the controlled release of BMP-2 and BMP-12, which contributes to the biological recovery process of bone and tendon cells.

In the literature, there are two genetic studies that are relevant to our study in terms of use of BMP-2 and BMP-12 and the repair of tissues [39, 40].

The first study [39] indicates that recombinant adenovirus BMP-2 and BMP-12 (Ad-BMP-2, Ad-BMP-12) formed tissue-like tendons/ligaments, unlike BMP-2, BMP-4, and BMP-7. This signifies that proteins in bone and cartilage healing have not been fully studied. In a rat model in which a bone defect was formed, it was studied whether tendon/ligament healing was the case by using plasmid vector coding BMP-12. In the study, 100 micrograms of BMP-2 or plasmid vector coding-12 was placed in lyophilized atelocollagen plates. Histological and molecular analyses of this drug delivery system were made in the second, fourth, and eighth weeks after it had been placed into bone defect in distal femoral metaphysis. It was found that significant fibrogenesis induction was observed in the second week, and bone formation was observed in the eighth week in the BMP-12 applied group. It was found that collagen-3 gene expression increased after the second week, which was followed by elastin and six-1 gene expression increase in the fourth week. It was maintained that genes necessary for tendon/ligament formation decreased in the eight week, which was followed by osteogenesis. Moreover, it was argued that the part that induced BMP-2 also quickly induced collagen type I and syntheses of bone-related ALP, such as ALP. Also, it was mentioned that such a tissue design in bones might be beneficial for periodontal tissue engineering [39].

The second study is Fu et al.'s study [40], which focuses on the transformation of Ad-BMP-12 and mesenchymal stem cells (MSC) in peripheral blood into a structure like a tendon/ligament. They used MSCs, which were obtained from peripheral blood of 3- or 4-month-old New Zealand rabbits in an in vitro culture environment. They assigned the group in which they did not transfect the adenoviral vector as the control group. They evaluated morphological changes by inverted light microscopy. Transfection activity and changes in green fluorescence protein expression were assessed by flow cytometry. At the end of 14 d, tendon/ligament-specific markers were evaluated by immunohistochemical and real-time chain reaction. They noted that peripheral blood commenced green fluorescence protein expression in MSCs 8 h after transfection, and clear morphology was seen in cells after 24 h. They reported that type 1 collagen and tendon/ligament-specific genes increased in the transfected group. It was emphasized that Ad-BMP-12 increased induction in fibroblasts like tendon/ligaments [40].

Kuroda et al. noted that BMP had different physiological effects; BMP-2 stimulated osteogenesis, and BMP-12 helped tendon/ligament-like tissues form. They monitored rats with bone deficits in femurs for eight weeks after the implantation of plasmid vectors coding BMP-2 or BMP-12. They reported that whereas BMP-12 stimulated fibrillogenesis as of the second week, it started bone formation as of the eighth week. They also maintained that BMP-2 stimulated osteogenesis for eight weeks [41].

Rodeo noted that cytokines, which are used in chemotaxis, proliferation, matrix synthesis, and cell differentiation, can be used in rotator cuff tendon–bone repair in which regeneration did not have a regular histology [42]. In a sheep infraspinatus model, they studied osteoinductive growth factors such as BMP-2 and BMP-7; transforming growth factors 1, 2, and 3; fibroblast growth factor; and the effects of BMP-12 on tendon–bone healing. They underlined that they induced bone formation and fibrocartilage formation in cling areas, which increased resistance to deficiency. They maintained that cytokines and growth factors in platelet-rich plasma content obtained from autologous blood could be used in biological rotator cuff repair [42].

Niu et al. designed copolymeric micelles, which allow controlled release in microsphere platforms, in order to control inflammation and provide simultaneous dentin regeneration. They embrued cattle serum albumin or BMP-2 into this two-layer drug delivery system, a chitosan-prophylactic acid base. They underlined that albumin, the executer of the release into the environment, and BMP-2 repressed inflammation and induced odontogenesis. They concluded that this method was applicable in treatment methods that required multidrug release [43].

Xu et al. underlined the importance of tissue engineering and gene release systems on bone healing, a difficult period in clinics, and argued that new treatment modalities could be developed. They examined whether arginine/chitosan/plasmid DNA nanoparticles led to osteogenic difference after transfecting osteoblastic primary cells and increasing BMP-2 synthesis by means of the design allowing controlled release. They reported that BMP-2 hit the peak on the seventh day, that its release volume was high, and that the release continued for 42 d. They showed that ALP levels increased by Alizarin red stain and that this design induced osteogenesis. They also indicated that arg-cs was a gene vector [44].

Ma et al. used composite hydrogels, which are comprised of polymeric particles, like polylactide co-glycolic, as BMP-2 delivery system. Thus, they maintained in vitro bioactivity of BMP-2, which is in its protein structure. They showed that it was biocompatible in vitro through biodegradability tests. They concluded that polylactide co-glycolic containing gel in cranial defects in significant sizes resulted in bone formation [45].

Despite all studies in the literature, it was found that no study is oriented at both tendon and bone repair using a drug delivery system in which BMP-2 and BMP-12 were subsequently released into the environment in combination. In this respect, our study pioneers in literature.

It was found that BMP-2, which was embedded in the first layer, and BMP-12, which was embedded in the second layer, allowed controlled release in the drug delivery system in this study. By this means, it was reported that anabolic reaction belonging to bone and tendon cells was induced, and osteogenesis and tenogenesis increased, in addition to osteoblastic and tenoblastic activities. Furthermore, our data indicated that experimental groups had more healthy cell proliferation when compared to the control group, and our results were proved to be statistically significant (*p* < 0.01).

As it is known, when similar studies in literature are reviewed, it is understood that these studies are carried out using commercial cell lines and/or animal tissues. As cell lines contain one type of cells, they do not host microenvironments, and their genotypes are modified by phenotypes, they cannot imitate natural tissues. On the other hand, since tissues in animal experiment settings do not have the same sensitiveness that human tissues have, the data acquired from this kind of setting may be misleading [18–20, 24, 46].

We did not use cell lines or culturized settings obtained from animal tissues; rather, we used primary cultures obtained from humans, so we are of the opinion that our results are reliable. However, it should be kept in mind that our experimental analyses pertain to a preliminary report in which an in vitro setting was used. Not having in vivo results is the limitation of our study.

It is known that newly formed twisted bone transforms into lamellar bone depending upon the increase in mineralization in the matrix, as well as ALP and bone-specific proteins during endochondral bone formation and intramembranous bone formation. As a result, it reaches ordinary levels of type 1 collagen and other protein levels by remodeling [47, 48].

In our research, we observed that ALP activity increased in bone cell culture samples, which includes hydrogel when compared to the control group without hydrogel. This increase was statistically significant (*p* < 0.01).

In conclusion, weak cross connections in sodium tetraborate and PVA-based drug delivery systems reinforced amylum separators. The biodegradation was adjusted to 21 days in this design. We found that controlled BMP-2 and BMP-12 releases occurred without damaging bioactivity in the areas where the system was applied.

Drugs and/or molecules that are protein or different peptide structures with short half-lives may decrease harmful effects of active substances, as they facilitate conduct in local areas, maintain pharmacologic levels at therapeutical levels, and target a certain cell or tissue. Moreover, it is advantageous in that therapeutic doses decreased to lower-than-needed levels.

We found that this design allowed controlled release as diffusion-controlled osmotic or swellable systems. We concluded that the number of healthy cells and proliferation might be superior to control groups by means of this biocompatible and biodegradable design, whose hydration rate was set (*p* < 0.01).

## 5. Conclusions

Our research is a preliminary report describing a study conducted in vitro. The same experimental setup, following the assessment of the biopolymeric prototype material by biomechanical tests, should be applied to live mammalians. We believe that such prototype systems may be pioneers in targeted drug therapies.

## Figures and Tables

**Table 1:** Statistical analyses; Tukey pairwise comparisons for MTT cell viability, toxicity, and proliferation (OD. 540 nm absorbance).

Time (day)	Group I	Group II	Group III	Group IV	*p* ^*∗*^
1	0,368 (F)^*¥*^	0,367 (F)^*¥*^	0,276 (F)^*¥*^	0,279 (F)^*¥*^	<0.01
7	0,412 (E)^*¥*^	0,517 (D)^*¥*^	0,396 (E)^*¥*^	0,594 (D)^*¥*^	<0.01
14	0,615 (C)^*¥*^	1,968 (A)^*¥*^	0,589 (D)^*¥*^	0,783 (B)^*¥*^	<0.01
21	0,917 (B)^*¥*^	2,042 (A)^*¥*^	0,675 (C)	1,173 (A)^*¥*^	<0.01

^*∗*^Analysis of variance (ANOVA); ¥: post hoc Tukey HSD test (Grouping information using the Tukey method and 95% confidence interval).

## Abbreviations

ALP: Alkaline phosphatase
BMP: Bone morphogenetic protein
DMEM: Dulbecco's modified eagle's medium
ELISA: Enzyme-linked immunosorbent assay
ESEM: Environmental scanning electron microscope
HSD: Tukey's honestly significantly different
MTT: (3-[4,5-Dimethylthiazol-2-yl]-2,5-diphenyltetrazolium bromide)
nm: Nanometer
OD: Optical density
PVA: Poly(vinyl) alcohol.

## Additional Points

*Availability of Materials*. Collagenase type I and II enzymes (1 mg/mL; Invitrogen Corporation) and HBSS (cat# 14025) were purchased from Gibco. Penicillin-streptomycin, fetal bovine serum, DMEM (1000 mg glucose/L), alpha-minimum essential medium eagle alpha modification (cat# M8042), and p-nitrophenyl phosphate substrate—which was used for ALP (IU/L) activity spectral measurements as a colorant (cat# N7653)—were supplied by Sigma Chemical, St. Louis, USA. An osteogenic stimulator kit (cat# 05434) containing MesenCult basal medium (cat# 05431), MesenCult Basal Medium (cat# 05435), and *β*-Glycerophosphate (cat# 05436) was supplied by Stemcell (Canada). Primary cell culture consumables were supplied by Sarsted Greigner. Transwell chambers were purchased from Corning Sigma (Transwell® polycarbonate membrane cell culture inserts CLS3422). An MTT (3-[4,5-dimethylthiazol-2-yl]-2,5-diphenyltetrazolium bromide) cell proliferation assay, a commercial kit that was used for cell vitality, toxicity, and proliferation, was supplied from Cell Biolabs Inc., USA (MTT cell proliferation assay cat# V-13154). A laminar flow cabin (Air Flow-NF–800 R) and incubator (06750) were sourced from Nuve, Ankara, Turkey. The inverted light microscope was an Olympus CKX41. The ELISA that was used in spectrophotometric measurements was a Mindray MR-96A, PRC. The ESEM was a Quanta FEG 250 from Fei.
